# Influence of care modes and social resources on psychotropic medication use in community-dwelling dementia patients

**DOI:** 10.3389/fpsyt.2023.1196801

**Published:** 2024-01-16

**Authors:** Wei-Chieh Chan, Wen-Fu Wang, Yu-Chun Tung, Ming-Che Chang, Hong-Ting Chan, Kai-Ming Jhang

**Affiliations:** ^1^Department of Neurology, Changhua Christian Hospital, Changhua, Taiwan; ^2^Department of Pharmacy, Taichung Veterans General Hospital, Taichung, Taiwan; ^3^Department of Nuclear Medicine, Changhua Christian Hospital, Changhua, Taiwan; ^4^Department of Long-Term Care Medicine, Puli Christian Hospital, Nantou, Taiwan

**Keywords:** psychotropic agent, dementia, community-dwelling, antipsychotic, antidepressant, sedative

## Abstract

**Background:**

Optimal use of psychotropic medications for people living with dementia is important. By finding potentially modifiable factors, dementia care teams may find solutions to achieve the appropriate use of psychotropic drugs.

**Objective:**

This study aimed to elucidate patient and caregiver factors associated with the use of psychotropic drugs listed in the potentially inappropriate medications (PIMs) in community-dwelling people with dementia.

**Methods:**

This cross-sectional study enrolled 808 patients newly diagnosed with dementia, and their caregivers, from a dementia clinic at Changhua Christian Hospital. Patient and caregiver characteristics, care mode, and social resource usage were recorded. Multivariate logistic regression was used to identify factors associated with prescribing psychotropic medications.

**Results:**

Of all the participants, 39.1% used at least one of psychotropic medication categorized as PIM. Patients with frontotemporal dementia, with behavior or psychological symptoms, or cared by sole foreign care workers; caregivers with higher depression scores, employed or non-spouse caregivers carried a higher risk of prescription of psychotropic medications listed in PIMs.

**Conclusion:**

Psychotropic drug prescriptions are associated with patient and caregiver factors. Therefore, implementing appropriate interventions, especially those targeting potentially modifiable factors, is important to reduce psychotropic medication use.

## Introduction

The prevalence of dementia is on a rapid incline, with an estimated 55 million individuals worldwide currently affected, and this number continues to rise (1). Progressive deterioration of cognitive function renders them dependent, and the intensity of care required increases substantially. In addition to diminished self-care abilities, many patients also experience neuropsychiatric symptoms, contributing to a heightened caregiver burden (2). Psychotropic drugs have been reserved for those with moderate to severe symptoms and at risk of self-mutilation or assault (3). Conversely, those who present with mild symptoms should be provided with nonpharmacological treatment, such as education to their caregivers (4).

Researches have shown that psychotropic drugs, especially antipsychotics, increase the incidence of falls, arrhythmias, metabolic syndrome, and the mortality rate in individuals with dementia (5). The US Food and Drug Administration has also recommended reducing antipsychotic use (6). The use of antidepressants in dementia patients improves agitation, but adverse effects such as hyponatremia should be taken in to consideration (7). Sedative hypnotics improve nighttime symptoms but may cause deterioration in cognitive function and increase the risk of falls (8). Therefore, ensuring the judicious and appropriate use of psychotropic drugs is crucial for effective dementia care.

Various studies have attempted to identify factors linked to the utilization of psychotropic drug in community-dwelling people with dementia. Both patient and caregiver characteristics are reportedly associated with psychotropic drug use. For example, Kindstedt et al. described psychotropic drugs and their related factors among older Swedish people with dementia (9). Female sex, older age, long time since diagnosis, and Lewy body dementia had a higher frequency of psychotropic drug prescription. Another study conducted in the US and Finland also revealed that older women with Alzheimer’s disease (AD) were more likely to use psychotropic drugs compared to men (10). Törmälehto et al. reported that increasing dependency, disease severity, and worse neuropsychiatric symptoms in the AD population correlated with psychotropic medication use in a three-year prospective study (11). Grace et al. concluded that caregiver characteristics, such as race and overall vigilance, were significantly associated with the use of psychotropic medications (12). Additionally, Acetylcholinesterase inhibitor use, especially at higher doses, may be associated with delayed initiation of antipsychotics and anxiolytics in Alzheimer’s dementia patients (13).

In Taiwan, research on dementia patients who use psychotropic drugs is relatively rare. Many factors, such as special care modes and community care facilities provided by the government, distinguish from in Western countries. These distinctive features might be connected to the patterns of psychotropic medication use. This study aimed to elucidate the factors associated with the utilization of psychotropic drugs in individuals with dementia. By identifying potentially modifiable factors, dementia care teams may find solutions and develop strategies to facilitate the appropriate use of psychotropic drugs.

## Methods

This cross-sectional study was conducted at the Dementia Center of Changhua Christian Hospital, a tertiary medical center in central Taiwan. The care team conducted face-to-face interviews with patients newly diagnosed with dementia, as well as their care partners. The interviews assessed cognitive function, living status, living environment, behavioral and psychological symptoms of the patient, care needs, burden, and the caregiver’s mood. A clinical pharmacist reviewed all medications to minimize drug interactions affecting cognitive function. All data, including medications used in the interviews, were recorded in electronic charts by case managers and pharmacists. Subjects assessed between October 2015 and September 2021 were included in the analysis.

The use of psychotropic drugs was further subdivided into the use of antipsychotics, antidepressants, and sedatives. We utilized Beer’s Criteria to differentiate potentially inappropriate medication (PIM) for elderly adult (14). All antipsychotics and sedatives are PIMs. Antidepressants with high cholinergic effect, including imipramine, amitriptyline, melitracen and paroxetine, are categorized as PIMs.

The National Institute on Aging-Alzheimer’s Association (NIA-AA) (15, 16), the International Society for Vascular Behavioral and Cognitive Disorders (VASCOG) (17), the Movement Disorder Society-Task force criteria (18), the Fourth Consensus Report of dementia with Lewy bodies (DLB) Consortium (19), and the International consensus criteria for behavioral variant FTD (20) were used for the diagnosis of AD, vascular cognitive impairment (VCI), Parkinson’s disease dementia (PDD), dementia of Lewy body (DLB) and FTD. Patients who met the NIA-AA criteria for AD and the VASCOG criteria for a possible major vascular cognitive disorder were classified as having mixed dementia.

This study was approved by the Institutional Review Board of Changhua Christian Hospital (CCH IRB 211104). The Institutional Review Board of CCH waived the need for informed consent because all data were extracted from electronic charts after the deletion of personalized information.

### Measurements

#### Measurement of patient features

Each patient’s sex, age, education level, underlying medical illness(es), ambulatory status, cohabitation status, resource utilization, and dementia subtype were collected at the initial assessment. Patient psychotic, behavioral, mood, and sleep symptoms, as well as the presence of pain, were also recorded by the case managers. The global clinical dementia rating scale (CDR) was used to assess dementia severity. The use of resources was classified into five groups: (1) not using any social resources, (2) daycare centers, (3) community aging care centers (Community Service Center for Dementia or community elderly stations), (4) home services (resident care attendants providing bathing or household chores at home), and (5) both services (use home and community services simultaneously). Activities of daily living (ADL) were categorized as dependent if any of the activities required assistance from others. The use of cholinesterase inhibitors and memantine was also recorded. Patients were categorized based on their use psychotropic drugs, including antipsychotics, antidepressants, and sedative medications.

#### Measurement of caregiver factors

Each caregiver’s age, marital status, employment, and relationship to the patient were recorded during the interview. The Zarit Burden Interview (ZBI) and the Center for Epidemiologic Studies Depression Scale (CES-D) were used to assess caregiver burden and mood status. There were five care modes in the study: Mode 0 (the patient’s ADL were independent, and the caregiver only accompanied the patient), Mode 1 (care by an informal solo caregiver), Mode 2 (care by more than two caregivers that could include a foreign care worker), Mode 3 (alternating care at different children’s homes), and Mode 4 (care by a sole foreign care worker).

### Statistical analyses

All data were analyzed using R software (R Foundation for Statistical Computing). Pearson’s chi-squared test or Fisher’s exact test was used to test for differences in the categorical data. Numerical data were tested using the Student’s t-test, Kruskal-Wallis rank sum test, or ANOVA test. Multivariate logistic regression models were applied to predict factors associated with the use of psychotropic drugs. Differences were considered statistically significant when the value of p was <0.05.

## Results

**Figure 1:**
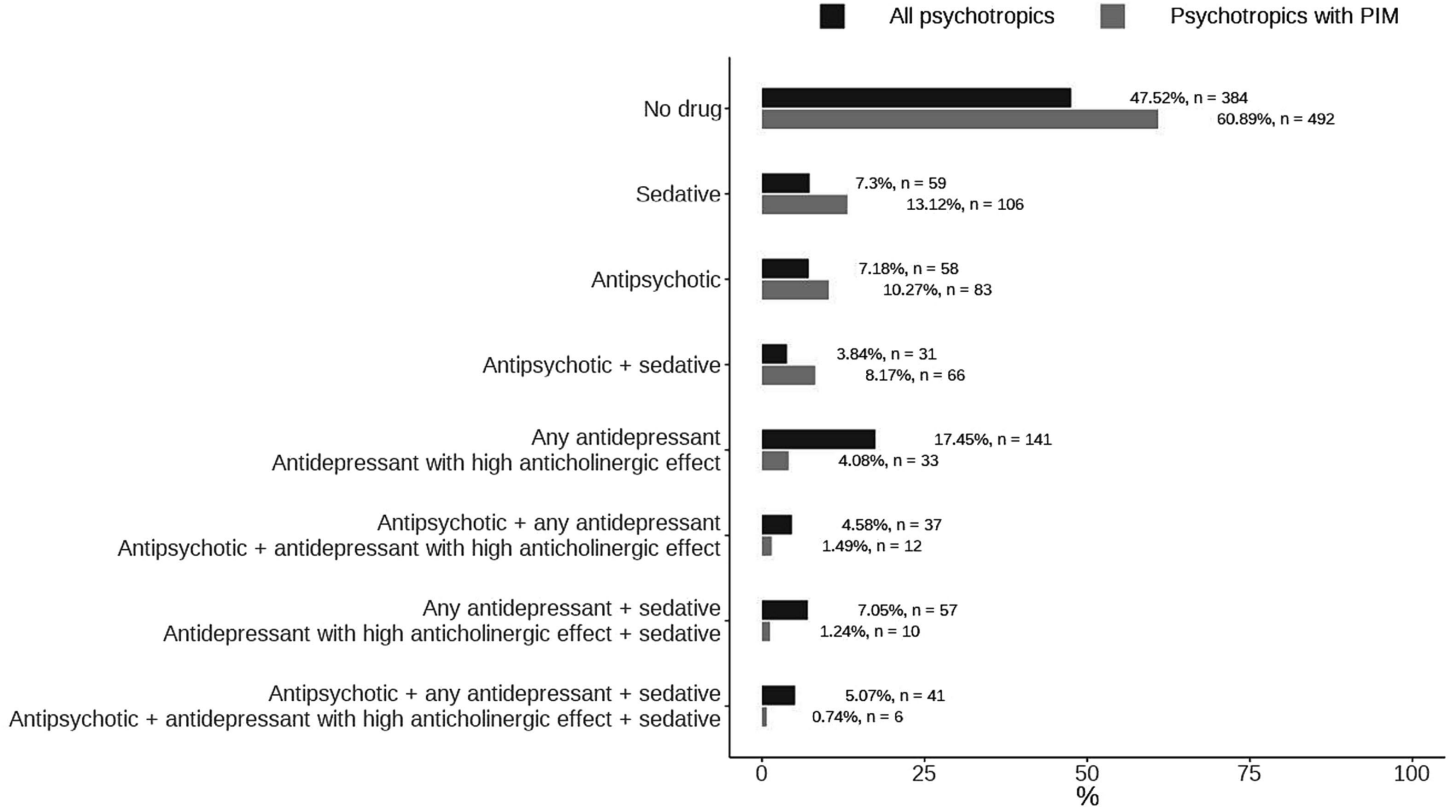
Distribution of different psychotropic drugs and psychotropic drugs listed in the PIM.

A total of 808 participants were recruited, of which 424 used psychotropic drugs and 316 used psychotropic drugs listed in the PIM. Figure 1 shows the distribution of different psychotropic drugs and psychotropic drugs which are listed in the PIM. 39.1% patients used PIMs. Most participants used only one kind of PIM, with 106(13.12%), 83(10.7%), 33(4.08%) used sedatives, antipsychotics, antidepressant with high anticholinergic effect, respectively. 6 participants (0.74%) used all three kinds of PIMs.

Table 1 shows the baseline characteristics of PIM users and non-users. The participants’ mean age was 79.80 ± 7.46 and 77.58 ± 8.90 years in PIM users and non-users, respectively. The gender distribution of patients was similar between the groups, with the male sex accounting for 32% of psychotropic drug users and 38% of non-users. Patients receiving psychotropic drugs are more likely to be more ADL dependent, be less likely live alone, have higher CDR score, less education years, and have more psychotic, behavioral, mood, or sleep problems. Caregivers of psychotropic users also had higher ZBI and CES-D scores and were more likely to be employed. Non-spouse informal caregivers or foreign care workers are more likely to care for psychotropic users.

**Table 1:** Characteristics of patients and caregivers using psychotropic drugs listed in the potentially inappropriate medications.

	Non-users *N* = 492	Psychotropic drug users *N* = 316	*p* value
**Patient factors**			
Male (%)	187 (38.0)	103 (32.6)	0.136
Age (SD)	77.58 (8.90)	79.80 (7.46)	<0.001
CDR (%)0.512 3	239 (48.6) 153 (31.1) 71 (14.4) 29 (5.9)	118 (37.3) 118 (37.3) 64 (20.3) 16 (5.1)	0.008
Education (SD)	5.77 (4.76)	4.88 (4.43)	0.008
Ambulation (%) Free Assisted device Wheelchair or bedridden	327 (66.5) 120 (24.4) 45 (9.1)	192 (60.8) 85 (26.9) 39 (12.3)	0.188
Diagnosis (%) AD VCI Mixed^a^ DLB PDD FTD Others	277 (56.3) 90 (18.3) 17 (3.5) 10 (2.0) 19 (3.9) 8 (1.6) 71 (14.4)	167 (52.8) 61 (19.3) 8 (2.5) 9 (2.8) 18 (5.7) 9 (2.8) 44 (13.9)	0.621
Disease (%) DM HTN Dyslipidemia CKD CVD CVA	151 (31.1) 297 (60.4) 183 (37.2) 48 (9.8) 35 (7.1) 63 (12.8)	113 (35.8) 187 (59.2) 135 (42.7) 44 (13.9) 25 (7.9) 46 (14.6)	0.194 0.793 0.135 0.088 0.776 0.545
ADL dependent (%)	110 (22.4)	102 (32.7)	0.002
Associated Symptoms^d^ (%) Pain Psychotic symptoms Behavioral symptoms Mood symptoms Sleep problem	40 (8.1) 106 (21.5) 39 (7.9) 197 (40.0) 103 (20.9)	39 (12.3) 121 (38.3) 63 (19.9) 182 (57.6) 122 (38.6)	0.065 <0.001 <0.001 <0.001 <0.001
Cohabitation (%) Live alone Spouse only Spouse/children Children only Others	30 (6.1) 109 (22.2) 164 (33.3) 161 (32.7) 28 (5.7)	18 (5.7) 64 (20.3) 84 (26.6) 117 (37.0) 33 (10.4)	0.040
Resources^b^ (%) No use Daycare center Community aging care centers Home services Both services	403 (81.9) 22 (4.5) 25 (5.1) 37 (7.5) 5 (1.0)	270 (85.4) 16 (5.1) 7 (2.2) 20 (6.3) 3 (0.9)	0.306
Allowance (%) No Government insurance Labor pension	166 (33.7) 175 (35.6) 151 (30.7)	115 (36.4) 103 (32.6) 98 (31.0)	0.641
Use ACHEI or memantine (%)	287 (58.3)	167 (52.8)	0.144
**Caregiver factors**			
Carer age (SD)	57.37 (13.92)	56.89 (12.59)	0.618
ZBI (SD)	25.80 (16.11)	29.52 (17.92)	0.002
CES-D (SD)	11.91 (10.24)	14.57 (11.01)	0.001
Widow or not-married (%)	93 (18.9)	56 (17.7)	0.742
Employed (%)	263 (53.5)	194 (61.4)	0.032
Relationship (%) Spouse Children Others	153 (31.1) 261 (53.0) 78 (15.9)	65 (20.6) 186 (58.9) 65 (20.6)	0.003
Care mode^c^ (%) Mode 0 Mode 1 Mode 2 Mode 3 Mode 4	120 (24.4) 169 (34.3) 160 (32.5) 6 (21.2) 37 (7.5)	44 (13.9) 105 (33.2) 117 (37.0) 6 (1.9) 44 (13.9)	<0.001

CDR = clinical dementia rating scale; AD = Alzheimer’s disease; VCI = vascular cognitive impairment; DLB=Dementia of Lewy body; PDD=Parkinson’s disease dementia; FTD = frontotemporal dementia; DM = diabetes mellitus; HTN = hypertension; CKD = chronic kidney disease; CVD = cardiovascular disease; CVA = cerebrovascular disease; CES-D = The Center for Epidemiologic Studies Depression Scale; ZBI = Zaria’s caregiver burden.

a Mixed type indicate mixed AD and VCI.

b Resources indicate the utilization status of social resources when ZBI was recorded. “Community aging care centers” include Community Service Center for Dementia or community elderly stations. “Home services” indicate bathing or household chores providing by resident care attendant. “Both services” indicate the subject use home and community services simultaneously.

c Care mode indicate most of the time how caregivers take care of people living with dementia. Mode 0 = ADL independent, caregivers only accompanied with the subject; Mode 1 = care by sole informal caregiver; Mode 2 = care by more than two caregivers (can include foreign care worker); Mode 3 = care at different child’s home alternately; Mode 4 = care by sole foreign care worker.

d Psychotic symptoms include delusion, hallucination, agitation or aggression, illusion; Behavioral symptoms include apathy, akathisia, disinhibition, scream or shouting, stereotype; mood symptoms include depression, anxiety, angry, and mood liability; Sleep problem include poor sleep onset, sleep interruption or irregular circadian rhythm.

Table 2 compares the characteristics of patients and caregivers among antipsychotic, antidepressant, antidepressant with high anticholinergic effect, and sedative users. Significant differences were observed in the age, CDR scores, ambulatory status, ADL dependency, and associated psychotic problems among antipsychotic, antidepressant, and sedative users. Antidepressant users tended to be younger, ADL-independent, and walk freely. Subjects with psychotic symptoms were more frequently prescribed antipsychotics. The caregivers of antipsychotic users had the highest burden of care.

**Table 2:** Characteristics of patients and caregivers using antipsychotics, antidepressants, antidepressant with high anticholinergic effect, and sedatives.

	Antipsychotic users N = 167	Antidepressant users N = 276	Antidepressant with high anticholinergic effect N = 61	Sedative users N = 188	*p* ^§^
**Patient factors**					
Male (%)	51 (30.5)	100 (36.2)	26 (42.6)	53 (28.2)	0.16
Age (SD)	80.8 (7.4)	77.7 (8.2)	77.15 (7.25)	79.9(7.0)	<0.01
CDR (%) 0.5 1 2 3	29.4(17.4) 83(49.7) 44(26.3) 11(6.6)	114(41.3) 112(40.6) 43(15.6) 7(2.5)	31 (50.8) 22 (36.1) 6 (9.8) 2 (3.3)	86(45.7) 58(30.9) 35(18.6) 9(4.8)	<0.01
Education (yrs)	4.88(4.7)	5.31(4.6)	5.14 (4.73)	4.71(4.26)	0.34
Ambulation (%) Free Assisted device Wheelchair or bedridden	94(56.3) 47(28.1) 26(15.6)	194(70.3) 61(22.1) 21(7.6)	44 (72.1) 14 (23.0) 3 (4.9)	117(62.2) 52(27.7) 19(10.1)	0.02
Diagnosis (%) AD VCI Mixed^¥^ DLB PDD FTD Others	89(53.3) 33(19.8) 5(3.0) 7(4.2) 11(6.6) 5(3.0) 17(10.2)	142(51.4) 54(19.6) 6(2.2) 5(1.8) 15(5.4) 11(4.0) 43(15.6)	28 (45.9) 14 (23.0) 1 (1.6) 0 (0.0) 1 (1.6) 13 (21.3) 4 (6.6)	103(54.8) 34(18.1) 4(2.1) 4(2.1) 12(6.4) 2(1.1) 29(15.4)	0.67
Disease (%) DM HTN Dyslipidemia CKD CVD CVA	58(34.7) 95(56.9) 68(40.7) 24(14.4) 13(7.8) 25(15.0)	78(2.3) 155(56.2) 106(38.4) 28(10.1) 15(5.4) 40(14.5)	23 (37.7) 38 (62.3) 28 (45.9) 8 (13.1) 1 (1.6) 9 (14.8)	60(31.9) 112(59.6) 80(42.6) 24(12.8) 15(8.0) 29(15.4)	0.35 0.76 0.66 0.39 0.48 0.96
ADL dependent (%)	72(43.1)	60(21.7)	11 (18.0)	56(29.8)	<0.01
Associated Symptoms* (%) Pain Psychotic symptoms Behavioral symptoms Mood symptoms Sleep problem	21(12.6) 90(53.9) 41(24.6) 97(58.1) 73(43.7)	27(9.8) 95(34.4) 46(16.7) 175(63.4) 91(33.0)	7 (11.5) 21 (34.4) 14 (23.0) 39 (63.9) 23 (37.7)	23(12.2) 57(30.3) 35(18.6) 107(56.9) 72(38.3)	0.59 <0.01 0.12 0.31 0.07
Cohabitation (%) Live alone Spouse only Spouse/ children Children only Others	7(4.2) 33(19.8) 46(27.5) 61(36.5) 20(12.0)	17(6.2) 60(21.7) 75(27.2) 97(35.1) 27(9.8)	3 (4.9) 16 (26.2) 22 (36.1) 16 (26.2) 4 (6.6)	12(6.4) 40(21.3) 47(25.0) 68(36.2) 21(11.2)	0.98
Resources^#^ (%) No use Daycare center Community aging care centers Home services Both services	140(83.8) 9(5.4) 4(2.4) 12(7.2) 2(1.2)	238(86.2) 14(5.1) 7(2.5) 14(5.1) 3(1.1)	52 (85.2) 3 (4.9) 2 (3.3) 2 (3.3) 2 (3.3)	159(84.6) 14(7.4) 3(1.6) 11(5.9) 1(0.5)	0.93
Allowance (%) No Government insurance Labor pension	59(35.3) 53(31.7) 55(32.9)	91(33.0) 102(37.0) 83(30.1)	26 (42.6) 18 (29.5) 17 (27.9)	67(35.6) 69(36.7) 52(27.7)	0.73
Use ACHEI or memantine (%)	85(50.9)	148(53.6)	27 (44.3)	106(56.4)	0.59
**Caregiver factors**					
Carer age (SD)	56.8(12.0)	55.91(12.84)	55.49 (11.70)	58.74(13.23)	0.06
ZBI (SD)	32.4(18.0)	29.41(17.01)	30.82 (19.77)	26.79(16.13)	<0.01
CES-D (SD)	15.5(10.1)	14.91(10.82)	15.51 (11.56)	13.31(10.62)	0.13
Widow or not-married (%)	28(16.8)	48(17.4)	13 (21.3)	28(14.9)	0.77
Employed (%)	104(62.3)	168(60.9)	41 (67.2)	113(60.1)	0.91
Relationship (%) Spouse Children Others	31(18.6) 103(61.7) 33(19.8)	72(26.1) 142(51.4) 62(22.5)	15 (24.6) 62 (52.5) 14 (23.0)	44(23.4) 109(58.0) 35(18.6)	0.24
Care mode^¶^ (%) Mode 0 Mode 1 Mode 2 Mode 3 Mode 4	18(10.8) 50(29.9) 68(40.7) 4(2.4) 27(16.2)	50(18.1) 99(35.9) 84(30.4) 8(2.9) 35(12.7)	10 (16.4) 22 (36.1) 23 (37.7) 2 (3.3) 4 (6.6)	23(12.2) 66(35.1) 69(36.7) 2(1.1) 28(14.9)	0.15

^§^*p* (*p*-value): comparison between antipsychotics, antidepressants, and sedatives.

Table 3 presents the multivariate logistic regression predictive factors associated with PIM user. Patients with hypertension and spouse as caregiver had a lower tendency to use psychotropic drugs. Patients with FTD, associated symptoms, cared by sole foreign care workers, or caregivers with higher CES-D scores, employed status were more likely to have PIMs prescribed.

**Table 3:** Multivariate logistic regression to predict factors associated with psychotropic drug use.

	Any Psychotropic drugs listed in PIM *N* = 316		Antipsychotic users *N* = 167		Antidepressant users *N* = 276		Sedative users *N* = 188	
**Patient factors**	OR (95% CI)	*p*	OR (95% CI)	*p*	OR (95% CI)	*p*	OR (95% CI)	*p*
Male	0.92[0.63;1.34]	0.66	0.79 [0.48;1.30]	0.35	1.14 [0.77;1.68]	0.53	0.72 [0.46;1.13]	0.15
Age	1.02[1.00;1.05]	0.07	1.04[1.00;1.07]	0.04	1.00[0.97;1.02]	0.76	1.02[0.99;1.05]	0.30
CDR (Ref: 0.5) 1 2 3	0.89[0.59;1.35] 0.72[0.37;1.38] 0.50[0.21;1.22]	0.59 0.32 0.13	3.25[1.87;5.63] 2.37[1.09;5.13] 1.80[0.64;5.05]	<0.01 0.03 0.26	1.34[0.88;2.06] 1.04[0.52;2.05] 0.36[0.12;1.03]	0.18 0.92 0.06	0.51[0.32;0.83] 0.53[0.25;1.11] 0.46[0.17;1.29]	0.01 0.09 0.14
Education (yrs)	0.99[0.95;1.03]	0.73	1.02[0.97;1.07]	0.44	1.00[0.96;1.04]	0.82	0.98[0.941.03]	0.44
Ambulation (Ref: free) Assisted device Wheelchair or bedridden	0.72[0.47;1.10] 0.70[0.37;1.33]	0.13 0.28	0.53[0.31;0.91] 0.55[0.27;1.13]	0.02 0.11	0.69[0.44;1.08] 0.69[0.35;1.38]	0.11 0.30	0.87[0.54;1.39] 0.64[0.31;1.32]	0.55 0.23
Diagnosis (Ref: AD) VCI Mixed^¥^ DLB PDD FTD Others	1.19[0.67;2.11] 0.49[0.17;1.41] 1.11[0.40;3.14] 1.37[0.64;2.92] 3.37[1.02;11.14] 1.22[0.71;2.13]	0.56 0.19 0.84 0.42 0.05 0.47	1.10[0.54;2.25] 0.43[0.12;1.52] 1.78[0.57;5.54] 1.29[0.56;3.10] 1.92[0.47;7.86] 1.00[0.48;2.10]	0.79 0.19 0.32 0.57 0.36 1.00	1.55[0.84;2.84] 0.75[0.24;2.39] 0.90[0.29;2.82] 1.38[0.63;3.01] 5.67[1.69;19.01] 1.68[0.95;2.95]	0.16 0.63 0.86 0.42 <0.01 0.07	0.88[0.45;1.72] 0.53[0.15;1.81] 0.76[0.22;2.62] 2.05[0.90;4.68] 0.82[0.16;4.29] 1.20[0.65;2.25]	0.71 0.31 0.66 0.09 0.81 0.56
Disease DM HTN Dyslipidemia CKD CVD CVA	1.37[0.96;1.96] 0.69[0.49;0.98] 1.39[0.98;1.96] 1.32[0.80;2.18] 0.92[0.49;1.74] 1.16[0.69;1.95]	0.08 0.04 0.06 0.27 0.81 0.57	1.29[0.82;2.02] 0.67[0.43;1.03] 1.48[0.96;2.29] 1.11[0.60;2.05] 0.72[0.33;1.59] 1.05[0.55;1.99]	0.28 0.07 0.08 0.75 0.42 0.89	0.78[0.54;1.14] 0.83[0.58;1.20] 1.05[0.73;1.50] 0.95[0.56;1.63] 0.54[0.28;1.13] 1.27[0.74;2.17]	0.20 0.32 0.80 0.85 0.11 0.38	0.86[0.57;1.29] 0.86[0.85;1.28] 1.20[0.81;1.77] 1.28[0.73;2.25] 1.05[0.52;2.13] 1.61[0.90;2.86]	0.46 0.46 0.35 0.38 0.89 0.11
ADL dependent	1.38[0.82;2.32]	0.23	1.62[0.91;2.88]	0.10	0.68[0.39;1.17]	0.16	1.29[0.71;2.33]	0.41
Associated Symptoms Pain Psychotic symptoms Behavioral symptoms Mood symptoms Sleep problem	1.12[0.66;1.89] 1.54[1.05;2.26] 1.95[1.18;3.22] 1.45[1.04;2.02] 1.87[1.30;2.69]	0.68 0.03 0.01 0.03 <0.01	1.04[0.56;1.95] 2.88[1.85;4.47] 1.76[1.02;3.04] 0.98[0.64;1.50] 1.89[1.22;2.93]	0.90 <0.01 0.04 0.93 <0.01	0.83[0.47;1.46] 1.17[0.79;1.74] 1.15[0.69;1.92] 2.72[1.92;3.85] 1.38[0.94;2.01]	0.52 0.44 0.60 <0.01 0.10	0.91[0.50;1.63] 0.82[0.52;1.28] 1.83[1.07;3.16] 1.54[1.05;2.26] 1.71[1.14;2.57]	0.74 0.37 0.03 0.03 0.01
Cohabitation (Ref: live alone) Spouse only Spouse/ children Children only Others	1.27[0.57;2.83] 1.07[0.50;2.27] 0.99[0.48;2.04] 1.92[0.78;4.71]	0.55 0.86 0.97 0.16	2.79[0.92;8.49] 2.35[0.81;6.85] 1.77[0.63;4.93] 3.06[0.94;9.92]	0.07 0.12 0.28 0.06	0.81[0.35;1.85] 0.73[0.34;1.58] 0.80[0.38;1.69] 1.43[0.57;3.57]	0.62 0.42 0.55 0.44	0.92[0.38;2.23] 0.75[0.33;1.74] 0.73[0.33;1.63] 1.52[0.58;3.99]	0.86 0.51 0.45 0.40
Resources (Ref: No use) Daycare center Community aging care centers Home services Both services	0.95[0.44;2.06] 0.45[0.18;1.13] 0.79[0.41;1.50] 0.50[0.09;2.67]	0.89 0.09 0.47 0.42	0.75[0.30;1.86] 0.61[0.18;2.00] 1.00[0.46;2.18] 0.70[0.09;5.68]	0.53 0.41 1.00 0.74	0.95[0.43;2.11] 0.38[0.14;1.00] 0.73[0.36;1.49] 0.82[0.16;4.28]	0.90 0.05 0.39 0.81	2.51[1.13;5.61] 0.32[0.09;1.12] 0.95[0.45;2.03] 0.30[0.03;2.76]	0.02 0.07 0.90 0.29
Allowance (Ref: No) Government insurance Labor pension	0.83[0.56;1.23] 1.07[0.71;1.60]	0.35 0.75	0.76[0.46;1.27] 1.18[0.71;1.95]	0.30 0.52	1.70[1.12;2.58] 1.38[0.91;2.11]	0.01 0.13	1.03[0.66;1.61] 0.83[0.52;1.32]	0.88 0.43
Use ACHEI or memantine	1.01[0.66;1.57]	0.95	0.93[0.54;1.59]	0.78	1.10[0.70;1.74]	0.68	1.14[0.69;1.89]	0.60
**Caregiver factors**								
Carer age	1.02[1.00;1.04]	0.09	1.01[0.99;1.04]	0.50	0.99[0.97;1.01]	0.16	1.03[1.01;1.06]	0.01
ZBI	1.00[0.98;1.01]	0.74	1.01[0.99;1.02]	0.50	0.99[0.98;1.01]	0.43	0.99[0.97;1.00]	0.10
CES-D	1.02[1.00;1.05]	0.02	1.01[0.99;1.04]	0.39	1.03[1.01;1.05]	<0.01	1.02[0.99;1.04]	0.12
Widow or not-married	0.87[0.55;1.36]	0.53	0.85[0.48;1.51]	0.58	0.71[0.44;1.14]	0.16	0.73[0.42;1.24]	0.24
Employed	1.48[1.03;2.14]	0.03	1.65[1.04;2.61]	0.03	1.21[0.83;1.77]	0.32	1.43[0.94;2.17]	0.09
Relationship (Ref: Spouse) Children Others	2.27[1.10;4.70] 2.74[1.15;6.51]	0.03 0.02	1.84[0.71;4.73] 1.59[0.51;4.99]	0.21 0.42	0.77[0.37;1.61] 1.27[0.54;3.03]	0.49 0.58	1.85[0.82;4.20] 1.99[0.77;5.16]	0.14 0.16
Care mode (Ref: mode 0) Mode 1 Mode 2 Mode 3 Mode 4	1.59[0.94;2.68] 1.54[0.93;2.54] 2.04[0.55;7.58] 2.06[1.04;4.08]	0.08 0.09 0.29 0.04	0.86[0.41;1.78] 1.15[0.58;2.30] 1.80[0.39;8.27] 1.18[0.50;2.79]	0.68 0.69 0.45 0.70	1.42[0.84;2.39] 1.14[0.68;1.90] 4.82[1.22;19.12] 1.79[0.88;3.65]	0.19 0.63 0.03 0.11	2.57[1.38;4.79] 2.42[1.32;4.43] 1.22[0.22;6.81] 3.37[1.56;7.28]	<0.01 <0.01 0.82 <0.01

Table 3 also shows the predictive factors for antidepressant, antipsychotic, or sedative medications. For antipsychotic drugs, patients requiring an assisted device are less likely to use such drugs; however, older patients or with mild or moderate dementia (versus CDR = 0.5) or who had employed caregivers tended to use more antipsychotics. For antidepressant drugs, patients who used community aging care centers showed a decreased tendency to use these medications, while patients with FTD, on government insurance, had caregivers with higher CES-D scores, and experienced alternating care at different children’s homes were more likely to use such medications. Regarding sedative drugs, patients with mild dementia (CDR:1) were less likely to use these drugs; however, participants using daycare centers, had elderly caregivers, or needed to be cared for by others favored the use of sedative drugs.

## Discussion

This study identified multiple factors contributing to the use of psychotropic agents, encompassing perspectives from both patients and caregivers. Patients with frontotemporal dementia, as well as those experiencing psychotic, behavioral, mood, or sleep symptoms, or cared sorely by foreign care worker, demonstrated a higher likelihood of requiring psychotropic drugs within the PIM list. Caregivers with higher depression scores, employed status, or non-spouse caregiver were more inclined to utilize psychotropic drugs.

Several findings from our study are comparable to those of previous studies. For instance, Kindstedt et al. found a strong association between Lewy body dementia, frontotemporal dementia, and antipsychotic use. (9) This association was not significant in the present study; however, higher psychotropic drugs use was observed in patients with FTD in our study. Given that this study enrolled patients newly diagnosed with dementia, antipsychotics may not be the primary choice for patients with DLB and FTD. Women were more likely than men to use psychotropic medications in US and Finnish cohorts (10) while our study did not identify a sex difference. Previous studies have indicated that more dependent ADLs and more severe CDR are associated with the use of antipsychotics. (11) Our study showed significantly increased antipsychotics prescription if the patients had mild to moderate dementia (CDR score 1 to 2), and trend in patients with severe dementia (CDR score 3). Patients using assisted device prescribed fewer antipsychotics when compared with those with unrestricted mobility. A review done by Mühlbauer et al. concluded that antipsychotics may reduce agitation in dementia. (21) If the patients’ ambulatory ability was hampered, the adverse effect of agitation, such as risk of self-mutilation or harm to others would be substantially reduced, thus decreasing the necessity for antipsychotics in this patient group. Furthermore, Grace et al. described that caregiver characteristics such as race and overall vigilance and patients with pain were significantly associated with psychotropic medication use. (12) Another Swedish study showed that acetylcholinesterase inhibitor (ACHEI) users had a lower risk of antipsychotic initiation. (13) Pain and ACHEI did not emerge as significant risk factors in the present study.

In this study, the different care modes between caregivers and patients played a significant role in influencing the administration of psychotropic drugs. Patients under the care of a sole foreign care worker exhibited the highest risk of receiving prescriptions for psychotropics with PIM. An intriguing discovery was that patients who experienced alternating care at different children’s homes had the highest risk of being prescribed antidepressants. This identification highlights the unique relationship between caregivers and patients in Taiwan, a country heavily influenced by the traditional Chinese culture. There is an old Chinese saying, “Have an elderly at home is like having a treasure.” Many Taiwanese individuals view placing elders in nursing homes as unkind and even unethical; thus, care modes such as alternating living at different children’s homes, occurring approximately 2–4 weeks have emerged to balance the burden of each caregiver while upholding the collective goal of caring for the older members of the community. Family dynamics and a lack of established environmental routines are potentially modifiable risk factors for behavioral and psychological symptoms of dementia. (22) The association noted in this study revealed an interesting perspective for further exploration by researchers and care teams, especially in the East Asian region. There is an opportunity to conduct additional research and formulate protocols aimed at minimizing the use of psychotropic drugs in response to these unique caregiving patterns.

Although most predictors discovered in this study were difficult to modify, certain factors may be adjustable and can be addressed by the dementia care team to mitigate the use of psychotropic drugs. The use of community aging care centers significantly decreased the use of antidepressants. The efficacy may be related to maintaining social connections in a familiar environment, an important element for the dementia population to maintain overall function (23). These resources should be introduced by dementia care teams. The employment status of caregivers and higher depression scores in caregivers were associated with an increased use of psychotropic drugs. Connecting the social welfare system to support caregivers, such as providing respite care and appropriate treatment for caregiver depression, is essential. Interestingly, spouse as caregivers rather than children or others was linked to a reduced use of any psychotropic drug with PIM. Few articles discuss the relationship of spouse caregiving and psychotropic drug use. Grace et al. reported relationship between caregiver and recipient did not associate with psychotropic medication use (12). However, in our clinical experience, the caregiver standpoint between spouse and children often differs. Spouses would often be more tolerant of their companions’ behavior, whereas children often emphasize on “more convenient” caring and favor medication to counter the patients’ “misbehaving.”

Factors associated with the use of psychotropic drugs in dementia patients living in nursing homes have also been reported. Hamada et al. discovered that severe dementia was associated with a higher use of antipsychotics, while older individuals (≥85 years) and those who were bedridden were exhibited a decreased use of antipsychotics (24). Higher levels of care dependency and permanent restlessness were positively correlated, whereas older age and male sex were negatively correlated with the prescription of psychotropic medications in a large cohort of nursing home residents in Germany and Austria (25). Another study conducted in nursing homes in Slovenia showed that female sex, younger age, permanent restlessness, dementia, depression, and an increased number of prescribed medications were associated with the use of psychotropic medications (26). In both community and nursing home studies, an inverse relationship between age and psychotropic drug use has been observed. While increased age was identified as a risk factor for antipsychotic prescription in our study and another study involving a community-dwelling dementia population (9), potential explanations include the preserved ambulatory capability in the community-dwelling population, which may be associated with more challenging behaviors, particularly in patients with severe dementia and advanced age. Prescribing antipsychotics has been a quality indicator for nursing home residents with advanced dementia (27), which may also influence the inverse relationship.

Our study boasts several strengths, such as a substantial sample size, comprehensive exploration of various factors concerning both patients and caregivers, and the incorporation of resources and diverse care modes. However, it is essential to acknowledge several limitations inherent in our research. Firstly, the data were sourced from a solitary tertiary center in central Taiwan, thereby constraining the broader applicability of our findings. Additionally, the dataset exclusively pertains to patients newly diagnosed with dementia, potentially encompassing individuals with milder conditions. As a cross-sectional study, a principal limitation lies in the inability to establish a temporal link between the outcome and exposure.

In summary, the prescription of psychotropic drugs is linked to a combination of patient and caregiver factors. Notably, protective factors include patients being cared for by spouses, while caregivers who are employed, exhibit higher depressive scores, or function as sole foreign care workers are at a heightened risk of prescribing psychotropic drugs. Implementing targeted interventions to modify these factors is crucial for minimizing the utilization of psychotropic medications.

## Data availability statement

The original contributions presented in the study are included in the article/supplementary material, further inquiries can be directed to the corresponding author.

## Ethics statement

This study was approved by the Institutional Review Board of Changhua Christian Hospital (CCH IRB 211104). The Institutional Review Board of CCH waived the need for informed consent because all data were extracted from electronic charts after the deletion of personalized information.

## Author contributions

All authors listed have made a substantial, direct, and intellectual contribution to the work and approved it for publication.
